# Beta oscillatory responses in healthy subjects and subjects with mild cognitive impairment^☆^

**DOI:** 10.1016/j.nicl.2013.07.003

**Published:** 2013-07-17

**Authors:** Bahar Güntekin, Derya Durusu Emek-Savaş, Pınar Kurt, Görsev Gülmen Yener, Erol Başar

**Affiliations:** a Brain Dynamics, Cognition and Complex Systems Research Center, Istanbul Kültür University, Istanbul 34156, Turkey; b Brain Dynamics Multidisciplinary Research Center, Dokuz Eylül University, Izmir 35340, Turkey; c Department of Neurosciences, Dokuz Eylül University, Izmir 35340, Turkey; d Department of Neurology, Dokuz Eylül University Medical School, Izmir 35340, Turkey; e İstanbul Arel University, Psychology Department, Turkey

**Keywords:** EEG, Event related oscillations, Evoked oscillations, Beta, Mild cognitive impairment, Oddball paradigm

## Abstract

The aim of the present study was to investigate the role of beta oscillatory responses upon cognitive load in healthy subjects and in subjects with mild cognitive impairment (MCI). The role of beta oscillations upon cognitive stimulation is least studied in comparison to other frequency bands. The study included 17 consecutive patients with MCI (mean age = 70.8 ± 5.6 years) according to Petersen's criteria, and 17 age- and education-matched normal elderly controls (mean age = 68.5 ± 5.5 years). The experiments used a visual oddball paradigm. EEG was recorded at 30 cortical locations. EEG-evoked power, inter-trial phase synchronization, and event-related beta responses filtered in 15–20 Hz were obtained in response to target and non-target stimuli for both groups of subjects. In healthy subjects, EEG-evoked beta power, inter-trial phase synchronization of beta responses and event-related filtered beta responses were significantly higher in responses to target than non-target stimuli (p < 0.05). In MCI patients, there were no differences in evoked beta power between target and non-target stimuli. Furthermore, upon presentation of visual oddball paradigm, occipital electrodes depict higher beta response in comparison to other electrode sites. The increased beta response upon presentation of target stimuli in healthy subjects implies that beta oscillations could shift the system to an attention state, and had important function in cognitive activity. This may, in future, open the way to consider beta activity as an important operator in brain cognitive processes.

## Highlights

• Beta responses were analyzed upon presentation of visual oddball stimuli.
• Healthy subjects and mild cognitive-impairment patients were included in the study.
• Target stimulation yielded higher beta responses than non-target stimulation.
• Beta power of patients did not differ between target and non-target stimulation.
• Increased event-related beta might be related to attention and cognitive activity.

## 1 Introduction

The present study has two major aims: Firstly, although several studies have already shown sensory and cognitive correlates of beta activity, we encountered almost no studies showing change of target and non-target responses in the oddball paradigm in mild cognitive impairment (MCI) patients and also in healthy subjects. Secondly, we also encountered no studies applying three mathematical methods (event related power, event-related phase locking, event related filtered oscillatory responses) to the analysis of beta responses. In the present study we show differential beta response between healthy subjects and MCI patients, and the results can therefore be considered as new candidate biomarkers in MCI and AD in addition to changes in other frequency bands (Başar, 2011; Yener and Başar, 2013; Yener et al., 2007, 2008, 2009).

In earlier studies, Karrasch et al. (2006), Kurimoto et al. (2012) and Missonnier et al. (2007) reported differing beta responses between healthy subjects and MCI patients. These studies, however, applied only event-related power analysis of beta oscillations and did not use the visual oddball paradigm.

The functional role of beta oscillatory responses seems to be less analyzed in comparison to other frequency bands (Engel and Fries, 2010; Huster et al., 2013). In a recent review Huster et al. (2013) pointed out that activity in the beta band has attracted attention only lately in context of response inhibition. Beta oscillatory responses have been classically considered to be related to somatosensory and motor functions (Pfurtscheller et al., 1996). On the other hand Wróbel (2000) showed that beta band activity reflects arousal of the visual system during increased visual attention. Increased beta band activity was registered in the occipital cortex of dog that expected a picture of a rewarding piece of meat (Lopes da Silva et al., 1970). Recent experiments also showed increased beta responses upon application of emotionally negative stimuli (Güntekin and Başar, 2007a, 2010a; Woodruff et al., 2011). The role of beta oscillatory responses upon cognitive stimulation was studied by several groups (Cacace and McFarland, 2003; Ishii et al., 2009; Kukleta et al., 2009a; Mazaheri and Picton, 2005) but no consistent findings were reported.

The oddball paradigm is a method used to analyze cognitive processes, and P300 response is an important component of such studies (Polich and Kok, 1995). A series of studies on local oscillatory dynamics showed that the major operating rhythms of P300 are mainly delta and theta oscillations (Başar et al., 2001; Başar-Eroglu et al., 1992; Demiralp et al., 1999; Karakaş et al., 2000; Kolev et al., 1997; Spencer and Polich, 1999; Yordanova et al., 2000). Prolongation of theta and alpha oscillations was described for the target stimuli (Başar et al., 1997, 1999; Başar-Eroglu et al., 1992; Demiralp and Ademoglu, 2001; Ishii et al., 2009; Öniz and Başar, 2009; Stampfer and Başar, 1985; Yordanova and Kolev, 1998). Furthermore, delta response oscillations are higher in response to target stimuli compared to non-target stimuli and simple sensory stimuli (Başar-Eroglu et al., 1992; Demiralp et al., 1999; Güntekin and Başar, 2010b). However, few studies have analyzed beta oscillatory responses in oddball paradigm (Cacace and McFarland, 2003; Ishii et al., 2009; Mazaheri and Picton, 2005). Therefore, the role of beta oscillations in response to oddball paradigm remains unclear. Kukleta et al. (2009a) investigated the beta (25–35 Hz) synchronization of epileptic patients with intracerebral electrodes upon stimulation in visual oddball paradigm. Subjects were asked to count target signals mentally, and to also press a button upon target stimulation. These authors reported increased beta synchronization in response to both target and non-target responses. Accordingly, they associated the increased beta synchronization with cognitive demands.

In recent years, our group published studies on electrophysiological bio-markers for mild cognitive impairment (MCI), Alzheimer's disease (AD) (Yener and Başar, 2010, 2013) and bipolar disorder (BP) (Başar and Güntekin, 2008, 2012; Özerdem et al., 2008, 2010, 2011, 2013). Persons with MCI commonly have mild problems performing complex functional activities such as managing finances or shopping. Even though these functions are achieved less efficiently, MCI subjects need minimal assistance for their functionality or do not lose their independence. They express concern regarding a change in their cognition and show objective evidence of impairment in one or more cognitive domains typically involving memory. This stage is generally considered the symptomatic, predementia phase of Alzheimer's disease (AD) (Albert et al., 2011). Our group (Yener et al., 2008, 2012) and others (Caravaglios et al., 2008; Polikar et al., 2007) showed decreased delta ERO in either visual or auditory paradigm, along with decreased event-related coherence in alpha, theta, and delta frequency ranges (Başar et al., 2010; Güntekin et al., 2008) in AD. As in AD, MCI patients showed decreased event-related delta oscillations in comparison to healthy controls upon application of target stimuli (Kurt et al., 2012; Yener et al., 2012).

### 1.1 Why do we try to discuss beta oscillations in MCI and Alzheimer's disease, and why do we compare with healthy subjects?

In the analysis of spontaneous EEG of Alzheimer's disease it was shown that the hallmark of EEG abnormalities in AD patients is a shift of the power spectrum to lower frequencies and a decrease in coherence of fast rhythms as it was reviewed by Jeong (2004). The review of Dauwels et al. (2010) also showed that three major effects of AD on EEG have been observed: slowing of the EEG, reduced complexity of the EEG signals, and perturbations in EEG synchrony. Yener and Başar (2013) examined whether it is possible to develop an ensemble of biomarkers for MCI and Alzheimer's disease. Amyloid PET, fluoro-deoxy-glucose PET, cerebrospinal fluid (CSF), and magnetic resonance imaging (MRI) have already been proposed as biomarkers for MCI and AD. However, dynamic biomarkers are also important for tracking the progression of disease, or for monitoring drug effects. EEG equipment is less costly than PET and MRI. EEG has also the advantage of being a non-invasive technique.

Our previous research showed that it might be possible to develop an ensemble of biomarkers for MCI and AD. Earlier findings for mild AD subjects indicated decreased delta responses either using visual or auditory oddball paradigm (Caravaglios et al., 2008; Polikar et al., 2007; Yener et al., 2008, 2012). Theta oscillatory responses displayed lower values of phase-locking in frontal area in AD in comparison to healthy subjects (Yener et al., 2007). Güntekin et al. (2008) and Başar et al. (2010) reported that the healthy control group showed significantly greater event-related coherence in delta, theta and alpha bands compared with de novo and medicated AD groups upon application of target stimuli. In contrast, almost no changes in event-related coherences were observed in beta and gamma frequency bands (Başar et al., 2010). Yener et al. (in press) analyzed the event related delta, theta, alpha oscillatory responses of MCI patients and compared them with healthy controls. In the manuscript by Yener et al. (in press) it was shown that delta oscillatory responses were significantly lower in MCI patients in comparison to healthy controls, there were no group differences in the theta and alpha frequency ranges.

We encountered three studies analyzing beta oscillatory responses in MCI and AD subjects (Karrasch et al., 2006; Kurimoto et al., 2012; Missonnier et al., 2007). None of these three studies used visual oddball paradigm in their experiments, and in two of these studies subjects were asked to press a button, which may cause ERD of beta oscillations due to motor response. Karrasch et al. (2006) and Kurimoto et al. (2012) used Sternberg's working memory task as stimulation, whereas Missonnier et al. (2007) used attentional detection task and two-back task. Missonnier et al. (2007) analyzed only the T_5_, P_3_, P_4_, and T_6_ electrodes in the 1000–1700 ms time window after stimulus onset. Their analysis revealed that two-back-related beta ERS measured at parietal electrodes was of lower amplitude in progressive MCI and AD cases compared with healthy controls and stable MCI cases. Karrasch et al. (2006) demonstrated that, during the encoding phase, the responses in the 10–20 Hz frequencies were characterized by ERS in the control group; on the other hand, the responses were characterized by ERD in the MCI group. In a MEG study, Kurimoto et al. (2012) reported that patients with AD showed reduced beta event-related desynchronization (ERD) in the right central area compared to controls upon presentation of Sternberg's working memory task, and that there were no significant differences between MCI patients and healthy controls.

In the present study, it was hypothesized that if beta responses are related to cognitive processes, target stimulation will elicit higher beta responses than non-target stimulation. Moreover, since MCI patients have mild cognitive deficits, their beta responses should be lower than those of healthy controls.

## 2 Method

### 2.1 Subjects

The study included 17 consecutive, community-dwelling subjects with the diagnosis of amnestic MCI (mean age = 70.8 ± 5.6 years) according to the Petersen's criteria (Petersen et al., 1999) recruited from the neurology outpatient memory clinic, and 17 age- and education-matched normal elderly controls (68.5 ± 5.5) recruited from various community sources. All participants provided written informed consent prior to voluntary participation. None of the healthy controls were consanguineous to the patients. Mean education level was 10.7 years in the MCI group and 10 years in the control group. There were 9 females in the healthy elderly group and 7 in the MCI group. All AD subjects underwent extensive cognitive- and complete neurological, neuro-imaging (MRI) and laboratory examination, including: blood glucose, electrolytes, liver and kidney function tests, full blood count, erythrocyte sedimentation rate, thyroid hormone, vitamin B12, HIV, and VDRL. The cognitive testing included episodic memory (Öktem verbal memory processes test), non-verbal memory (Wecshler visual reproduction test), attention (WMS-R digit span test), orientation, executive functions (Stroop test, clock-drawing test, verbal fluency test), language (Boston naming test), and the Mini-Mental State Examination (MMSE). The patients diagnosed with MCI had subjective memory complaints verified by a relative, in addition to memory test score of 1.5 SD less than the mean age norm, and a CDR of 0.5, not reaching dementia criteria. Depressive co-morbidity was excluded on the basis of a geriatric depression scale score greater than 11 (GDS, Yesavage et al., 1983).

The group characteristics are shown in Table 1. Subjects with abnormal laboratory results indicating other causes of memory disorder, and those with vascular lesions on MRI were excluded from the study. All participants had normal vision, and none reported a history of head injury, or any other neurological or psychiatric disorders. All participants with regular use of anti-dementia drugs, antidepressants, neuroleptics, anti-epileptic medications, stimulants, opioids, or b-blockers were excluded from the present study. Informed consent was obtained from all subjects or their relatives. The present paper analyzed comprehensively the beta oscillatory responses in MCI patients and healthy controls by three different methods (ERSP, phase locking and filtered oscillatory responses). We analyzed delta, theta and alpha oscillatory responses of the same group of subjects in a separate paper (Yener et al., in press).

### 2.2 Stimulation

The participants sat in a dimly lit, isolated room during recordings. Classical visual oddball paradigm was applied using a simple 10-cd/m^2^ luminance light as the standard and a 40-cd/m^2^ luminance light as the target stimuli. The light appeared at full size on a 19-inch computer monitor with a refresh rate of 60 Hz. The duration of the stimulation was 1000 ms, the probability of the deviant stimuli was 0.33 and, in all paradigms, targets were embedded randomly within a series of standard stimuli. These stimulation signals were applied randomly, with inter-stimulus intervals varying between 3 and 7 s. In order to assess focused attention and working memory, the task required mental counting of the target stimuli.

### 2.3 Electrophysiological recording

EEG was recorded with 30 Ag/AgCl electrodes mounted in an elastic cap (Easy-cap) according to the international 10–20 system. Additionally, two linked earlobe electrodes (A1 + A2) served as references. The EOG from the medial upper- and lateral orbital rim of the right eye was also registered. For the reference electrodes and EOG recordings, Ag/AgCl electrodes were used. All electrode impedances were less than 10 kΩ. The EEG was amplified by means of a Brain Amp 32-channel DC system machine with band limits of 0.01–250 Hz. The EEG was digitized on-line with a sampling rate of 500 Hz.

Artifacts were eliminated manually and off-line, taking into consideration the EOG recorded from the right eye. The epoch numbers were equalized randomly between the target and non-target visual stimulation conditions.

### 2.4 Data analysis

#### 2.4.1 Event-related spectral perturbations

The epoching of continuous EEG files and artifact-related processing was performed with a BrainVision Analyzer (Brain Products GmbH). Single-trial epochs were extracted from − 500 to 1000 ms relative to stimulus.

For the analysis of the event-related spectral perturbations (ERSP) of beta responses, a Morlet-based wavelet transform with a ‘width’ of 6 cycles was employed (14 through 28 Hz) in order to provide a continuous measure of the amplitude of a frequency component between − 500 and 1000 ms (EEGLAB, Delorme and Makeig, 2004). Event-related spectral perturbations were computed on the wavelet-transformed epochs for each stimulus condition at each time point and wavelet frequency to yield time–frequency maps. To visualize magnitude changes in relation to a pre-stimulus baseline across the frequency range, we subtracted the mean baseline log spectrum (− 300 to − 50 ms) from each spectral estimate, producing the baseline-normalized time-frequency distribution. The color at each image pixel indicates amplification or attenuation (in dB) at a given frequency and latency relative to the time-locking event.

**Fig. 1:**
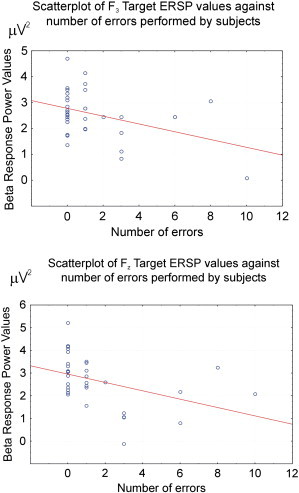
Scatter plot of F_3_ and F_z_ ERSP values against number of errors performed by subjects.

The peak amplitude/power at 15–20 Hz individual frequency was extracted for statistical assessment (Herrmann et al., 2004; Lenz et al., 2010, 2011). The grand average of ERSP of 17 healthy subjects and 17 MCI patients showed that event-related beta power was more pronounced in the 15–20 Hz frequency band (Fig. 1). Accordingly, we used each participant's peak amplitude/power at 15–20 Hz individual frequency. The peak-frequency was defined as the frequency bin showing the highest response in the time interval between 0 ms and 200 ms, obtained via time–frequency plots of the average response for each electrode and each subject.

#### 2.4.2 Inter-trial coherence (ITC)

Inter-trial coherence (ITC) is a frequency-domain measure for the synchronization of activity at a particular latency and frequency for a set of experimental events to which EEG data trials are time-locked (Delorme and Makeig, 2004; Tallon-Baudry et al., 1996). Here, we calculated the ITC using EEGLAB (see Delorme and Makeig, 2004) as follows:

For *j* = 1 to *N* trials,

$$\mathrm{ITC}\left(t,f\right)=\left\|\frac{1}{N}\sum_{i=1}^{N}e^{j\Phi_{i}\left(t,f\right)}\right\|$$

where Ф*_j_*(t, f) is the phase of the wavelet at time t and frequency f. ITC values range from 0 (indicating absence of phase-locking) to 1 (indicating perfect phase synchronization). All ITC values were baseline-corrected over − 300 ms to − 50 ms and were computed for each participant for grand average, ITC values were averaged across all participants. We used each participant's individual peak-frequency in the beta-band for the wavelet transform within the range of 15 to 20 Hz in the time interval between 0 ms and 200 ms.

#### 2.4.3 Digitally filtered event-related beta oscillatory responses

Digital filtering of ERPs was performed with Brain Vision Analyzer (Brain Products GmbH). The grand average of ERSP of 17 healthy subjects and 17 MCI patients showed that the event-related beta power was more pronounced in the 15–20 Hz frequency band (Fig. 1) Accordingly, each subject's averaged event-related potentials were digitally filtered in the 15–20 Hz frequency range. The maximum peak-to-peak amplitudes for each subject's averaged beta (15–20 Hz) responses were analyzed; that is, the largest peak-to-peak value in these frequency ranges in terms of μVs found in the time window between 0 and 300 ms.

#### 2.4.4 Statistical analysis

All statistical analyses were calculated using Statistica software. The differences between modalities were assessed by means of repeated measures of ANOVA. Three different measures (ERSP, ITC, filtered event-related beta responses) were analyzed separately. In the analysis, repeated measures of ANOVA included the between-subjects factor as group (healthy elderly controls, MCI patients); and the within-subject factors as stimulation (target vs. non-target) × 4 anterior-to-posterior (frontal, central, parietal, occipital) × 3 coronal (left medial, medial, right medial). Greenhouse–Geisser corrected p-values were reported. Post-hoc comparisons were analyzed with Bonferroni test. The significance level was set to p < 0.05 for all comparisons.

We also ran another ANOVA to compare the stimulation effect (target vs. non-target) in healthy subjects and in MCI subjects separately. In the analysis, repeated measures of ANOVA included the within-subject factors as stimulation (target vs. non-target) × 4 anterior-to-posterior (frontal, central, parietal, occipital) × 3 coronal (left medial, medial, right medial). Greenhouse–Geisser corrected p-values were reported. The significance level was set to p < 0.05 for all comparisons.

Pearson's correlation analysis was used to determine the correlation between beta responses and the number of errors performed by the subjects during mental count of the target stimuli.

## 3 Results

### 3.1 Behavioral results

In each measuring session, there were in total 40 target stimulation. Ten of the healthy control subjects counted the target stimulation as 40; five of the healthy subjects made one mistake while counting the target stimulation; and two of them made more than one mistake. Seven of the MCI patients counted the target stimulation as 40; three of the MCI patients made one mistake; and seven of them made more than one mistake. Pearson's correlation analysis showed that beta power was negatively correlated with the increasing number of mistakes at F_3_ (p < 0.02) and F_z_ (p < 0.02) locations upon presentation of target stimulation (Fig. 1). These results indicated that the subjects who made more mistakes had lower beta power at the defined electrode sites. There were no significant correlations between the number of mistakes and either beta-phase locking or filtered beta responses.

### 3.2 Results of event-related spectral perturbations

**Fig. 2:**
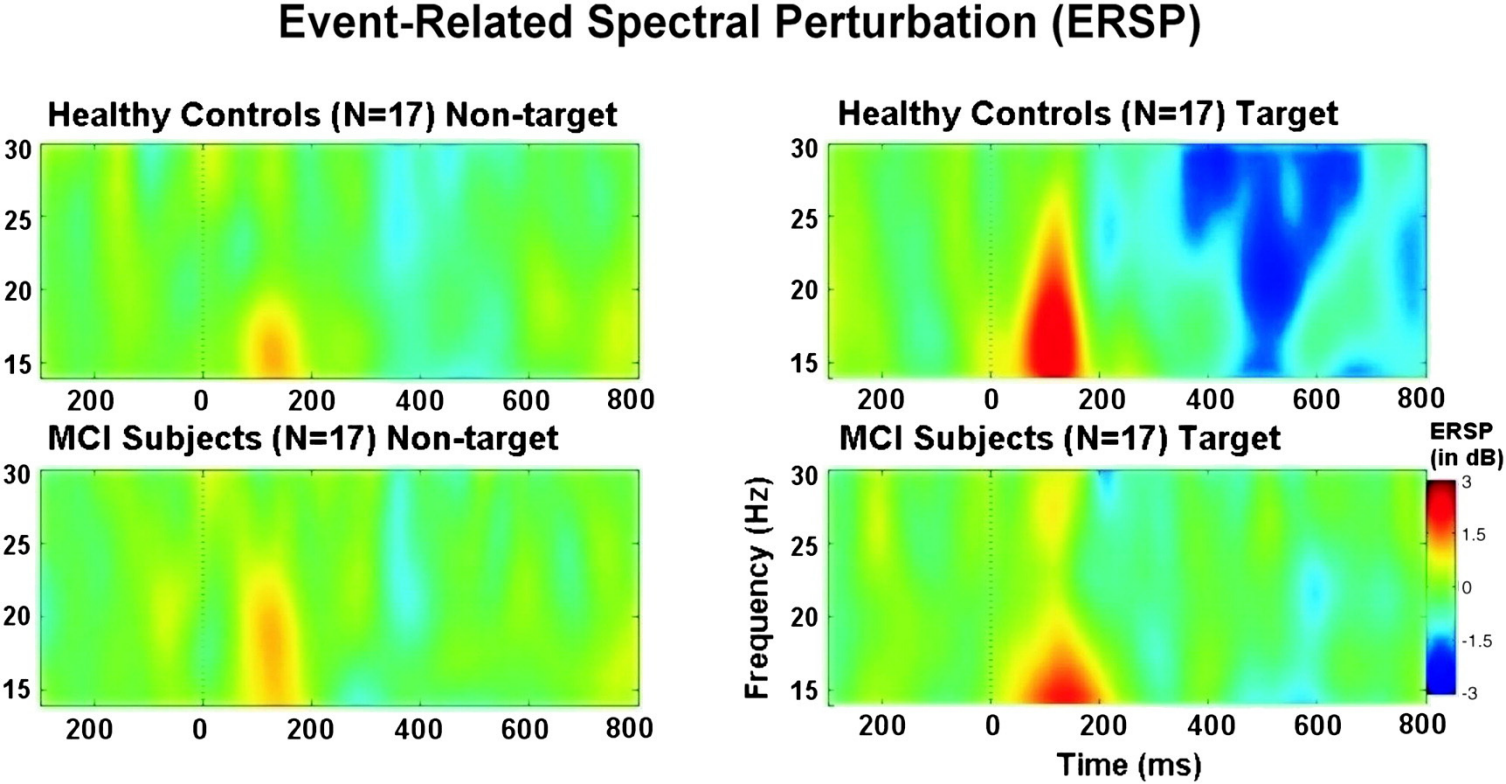
Grand average of event-related spectral perturbation (ERSP) of 17 healthy subjects and 17 MCI patients upon application of target (right frame) and non-target (left frame) stimulation.

Fig. 2 illustrates the grand average of time–frequency planes showing the post-stimulus enhancement of beta responses in both stimulations (target and non-target) for healthy controls (*N* = 17) (upper frame) and for MCI patients (*N* = 17) (lower frame) at F_4_ location. The grand average plots of ERSP analysis of targets and non-targets revealed that in the early time window (0–200 ms), the target stimuli elicited larger beta responses than non-target stimuli in both healthy controls and MCI patients. This differentiation was more pronounced for the healthy controls at all electrode sites. Furthermore, the grand average of ERSP showed that healthy subjects had a trend for higher beta responses than MCI patients at all electrode sites upon presentation of target stimuli. As an example, location F_4_ is presented in Fig. 2.

Statistical results for 2 groups × 2 stimulation × 4 anterior-to-posterior × 3 coronal were as follows: Although visual inspection of grand averages in all electrode sites revealed higher beta response in healthy elderly controls compared to MCI patients, this difference was not statistically significant. Within-subjects repeated measures of ANOVA revealed a significant difference for stimulation type [F(1,32) = 4.84; p < 0.04]. The post-hoc comparisons revealed that beta response power was significantly greater for target responses than for non-target responses. ANOVA of beta responses revealed significant results for coronal [F(2,64) = 4.14; p < 0.03] and for stimulation × anterior–posterior × coronal sites [F(6,86) = 3.79; p < 0.002]. Post-hoc comparisons showed that right electrode sites had higher values than the left electrode sites (p < 0.02). Post-hoc comparisons also showed that the differences between target and non-target responses were mostly significant at F_z_, F_4_, C_z_, C_4_, Oz and O_2_ electrode sites.

We also used ANOVA to compare only the stimulation effect (target vs. non-target) in healthy subjects and in MCI subjects separately. Repeated measures of ANOVA showed that in healthy controls, larger beta amplitude enhancement was elicited by targets when compared to non-target stimuli [F(1,15) = 6.66; p < 0.03]. On the other hand, there were no difference between target versus non-target beta responses in MCI patients in ERSP [F(0,15) = 0.424; p = 0.52].

### 3.3 Results of event-related inter-trial coherence

**Fig. 3:**
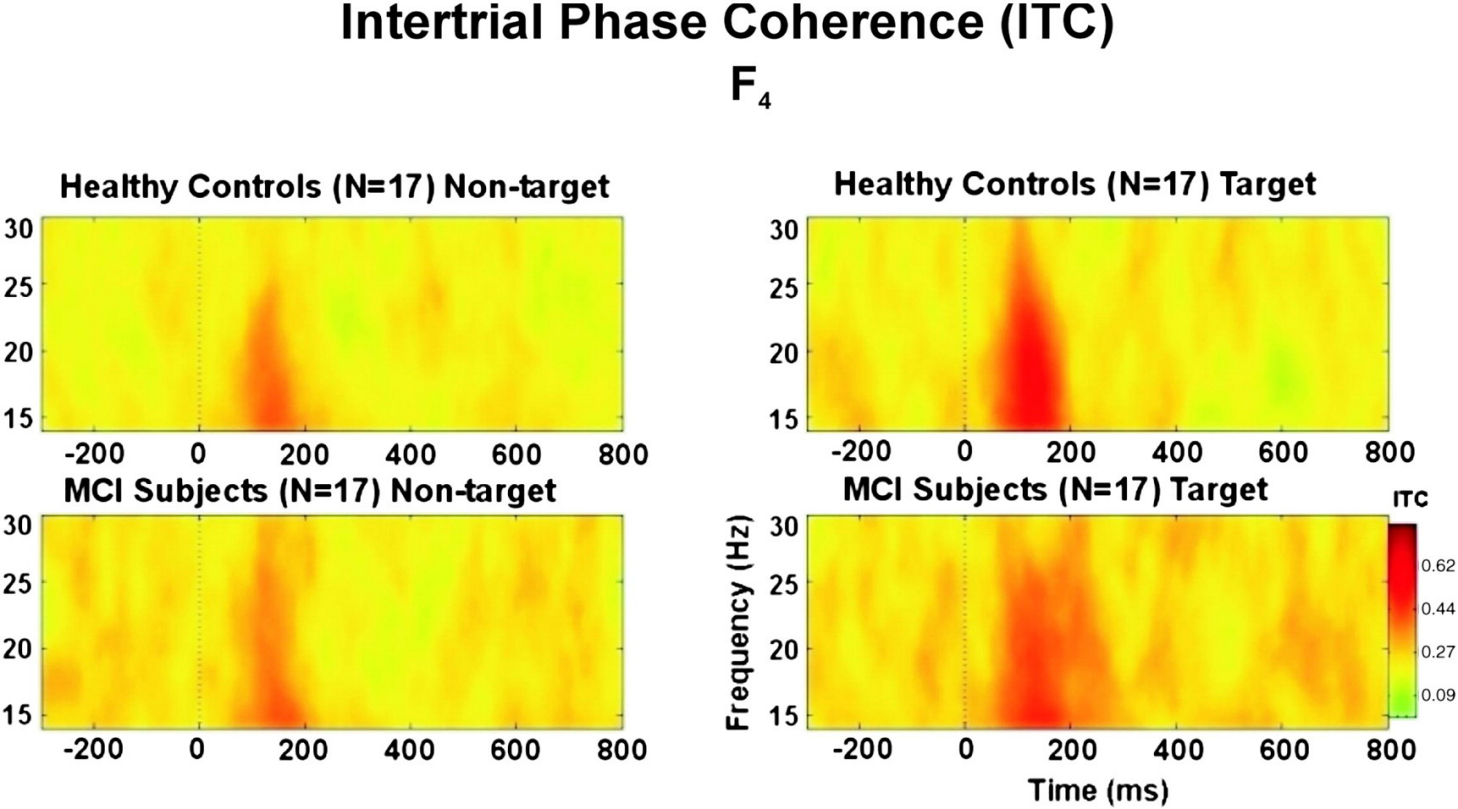
Grand average of inter-trial phase coherence of 17 healthy subjects and 17 MCI patients upon application of target (right frame) and non-target (left frame) stimulation.

Fig. 3 illustrates the grand average of time–frequency planes showing the inter-trial coherence of beta responses in both stimulations (target and non-target) for healthy controls (*N* = 17) (upper frame) and for MCI patients (*N* = 17) (lower frame) at location F_4_. The grand average plots of ITC analysis of targets and non-targets revealed that, in the early time-window (0–200 ms), target stimulation elicited approximately 50% greater beta-phase locking than non-target stimulation in both healthy controls and MCI patients. This differentiation is more pronounced for the healthy controls at all electrode sites. Furthermore, the grand average of ITC showed that healthy subjects had a trend for higher beta-phase locking than MCI patients at all electrode sites upon presentation of target stimuli.

Although beta inter-trial coherence measures were higher in healthy subjects than MCI patients, the difference was non-significant. Repeated within-subjects measures of ANOVA revealed a significant difference for stimulation type [F(1,32) = 24.87; p < 0.0001]. The post-hoc comparisons revealed that beta response power was significantly higher for target responses than for non-target responses. ANOVA of beta responses revealed significant results for coronal [F(2,64) = 5.00; p < 0.01] and for anterior–posterior [F(3,96) = 4.23; p < 0.0008] sites. Post-hoc comparisons showed that right electrode sites had higher values than the left electrode sites (p < 0.0008). Post-hoc comparisons also showed that occipital electrodes showed increased beta-phase locking in comparison to frontal (p < 0.05) and central (p < 0.008) electrodes.

### 3.4 Results of digitally filtered event-related beta (15–20 Hz) response oscillations

**Fig. 4:**
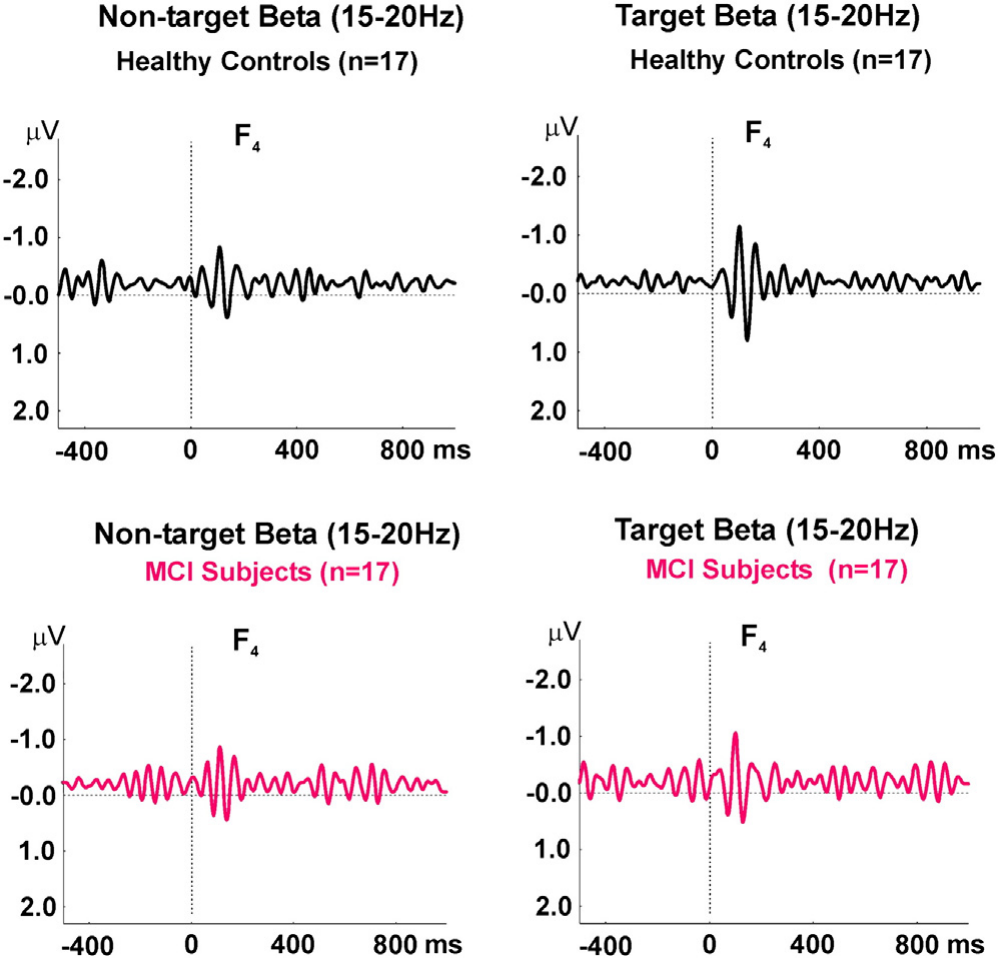
Grand average of filtered (15–20 Hz) beta oscillatory responses of 17 healthy subjects and 17 MCI patients upon application of target (right frame) and non-target (left frame) stimulation.

Fig. 4 illustrates the grand averages of beta responses in F_4_ electrode site upon presentation of target and non-target stimulation. As shown in Fig. 3, the grand averages of beta responses to target pictures are at least 53% higher than the grand averages of beta responses to non-target responses in healthy subjects. On the other hand, the grand averages of beta responses to target pictures are only 23% higher than the grand averages of beta responses to non-target responses in MCI patients. The grand average of beta responses showed that healthy subjects had a trend for higher beta responses than MCI patients at all electrode sites upon presentation of target stimuli. As an example, location F_4_ is presented in Fig. 3.

Although 15–20 Hz filtered beta responses were higher in healthy subjects in comparison to MCI patients, this difference was non-significant. Within-subjects repeated measures of ANOVA revealed a significant difference for stimulation type [F(1,32) = 37.01; p < 0.0001]. The post-hoc comparisons revealed that beta response power was significantly higher for target responses than for non-target responses. ANOVA of beta responses revealed significant results for coronal [F(2,64) = 3.77; p < 0.02]; for stimulation × anterior–posterior [F(3,96) = 3.40; p < 0.04] and for stimulation × anterior–posterior × coronal [F(3,96) = 2.43; p < 0.04]. Post-hoc comparisons showed that right electrode sites had higher values than the middle electrode sites (p < 0.03). Statistical analysis showed that target responses were significantly greater than non-target responses at all electrode sites. Post-hoc comparisons also showed that the differences between target and non-target responses were most pronounced at occipital electrode sites (p < 0.0001) and were highest at the right occipital (O_2_) (p < 0.0001) location.

## 4 Discussion

To our knowledge, the present study is the first to compare event-related beta oscillations in healthy subjects and MCI patients upon presentation of a visual oddball paradigm. In order to avoid movement-related responses, the subjects were asked to mentally count target stimuli. The results clearly demonstrated that target stimulation elicited higher event-related beta power, event-related beta-phase locking and event-related (filtered) beta responses in comparison to non-target stimulation at all electrode sites regardless of group effect. Furthermore, in healthy subjects, event-related beta power was higher upon presentation of target than non-target stimulati. Conversely, MCI patients displayed no difference. The results support the hypothesis that the increase of beta responses is also related to attention and the cognitive process.

### 4.1 Previous findings of beta oscillatory responses in movement-related, sensory, cognitive or emotional stimulation

Beta oscillatory responses are considered to be related to sensorimotor functions (Engel and Fries, 2010), and were decreased by voluntary movement (Pfurtscheller and Berghold, 1989; Pfurtscheller et al., 1996) and also by motor imagery (Neuper et al., 2009). Traub et al. (1999) showed that when the stimulation was intense the action potential burst depicts a transition from gamma frequency to beta frequency. Traub et al. (1999), Haenschel et al. (2000), Kisley and Cornwell (2006) concluded that beta band activity is closely related to stimulus-driven salience. Leventhal et al. (2012) suggested that beta oscillations reflect a post-decision stabilized state of cortical–basal ganglia networks, which normally reduces interference from alternative potential actions.

Several studies have shown that evoked oscillatory beta activity over scalp regions was associated with projections from sensory specific cortex. While auditory stimuli enhanced beta responses at central and temporal electrodes (Haenschel et al., 2000; Makinen et al., 2004; Peterson and Thaut, 2002), visual stimuli enhanced beta responses at occipital electrodes (Senkowski et al., 2006). Increased beta responses were also measured during multisensory stimuli in comparison to unisensory stimuli (Sakowitz et al., 2005; Senkowski et al., 2006).

Beta oscillatory responses were also related to emotional processes. In our previous study, increased left-frontal- and central beta-responses to “angry” face expression stimuli in comparison to “happy” face expression stimuli were reported (Güntekin and Başar, 2007a). Furthermore, female subjects had higher occipital beta responses in comparison to male subjects upon presentation of face expression paradigms (Güntekin and Başar, 2007b). In a recent study, we also demonstrated that negative valence images from the International Affective Picture System (IAPS) induced greater beta responses for negative as compared with positive images in frontal, central, and parietal electrodes (Güntekin and Başar, 2010a). These results were also supported by another group; Woodruff et al. (2011) demonstrated that negative valence IAPS imagery induced more beta power than neutral images did. Increased beta response during emotional pictures was not reported only at local circuits but also in long-range circuits. Miskovic and Schmidt (2010) examined coherences during affective image presentation and found an increase in beta responses during free viewing of high (pleasant and unpleasant) and low (neutral) emotionally arousing images. These studies merit additional attention, since their findings indicate that—independent of stimulus type (face expression or IAPS pictures)—pictures with negative emotions trigger increased beta responses in humans. Furthermore, this finding supports claims that neural activity in the beta range relates to processing of emotional stimuli (Güntekin and Başar, 2007a, 2010a; Miskovic and Schmidt, 2010; Woodruff et al., 2011).

In working memory paradigms divergent results were reported. Some studies showed increased beta responses (Kukleta et al., 2009a, 2009b; Onton et al., 2005; Ravizza et al., 2005; Tallon-Baudry et al., 1998), while others reported event-related desynchronization of beta responses (Cacace and McFarland, 2003; Ishii et al., 2009; Mazaheri and Picton, 2005). Voluntary movement causes ERD of beta responses (Babiloni et al., 2002; Pfurtscheller et al., 1996). The studies analyzing cognitive processes using button press showed beta ERD (Cacace and McFarland, 2003; Ishii et al., 2009; Karrasch et al., 2006; Mazaheri and Picton, 2005). In order to avoid effects of motor responses, Missonnier et al. (2007) analyzed only non-target responses, in which subjects did not press button; these authors reported ERS upon presentation of non-target stimuli. Kukleta et al. (2009a) required the subject to press a button in response to target detection and also to mentally count target stimuli. These authors reported increased beta activity in response to both target and non-target stimulation. As presented in this study and in the studies by Missonnier et al. (2007) and Kukleta et al. (2009a), if there is no button-press effect, then beta responses increase with cognitive load.

### 4.2 What do the results of the present study add to the literature?

Our study showed differing beta oscillatory responses between healthy controls and MCI patients in an early time window (0–300 ms) upon application of visual oddball paradigm. MCI patients have a trend to display lower event-related beta response than healthy controls. Although this difference did not reach a significance level in the group comparisons, there were differences between healthy controls and MCI patients in the identification of target stimulation versus non-target stimulation. In healthy subjects, target-event-related beta power was significantly higher than non-target beta power, whereas in MCI patients there was no such difference. Further, more extensive studies with a longitudinal design will be helpful to identify the differences between stable MCI, progressive MCI, and AD. There would likely be progressive decrease in event-related beta power and beta synchronization, from stable MCI to progressive MCI and to AD, upon application of cognitive stimulation.

The present study is the first to analyze event-related beta oscillations, in healthy subjects and MCI patients upon application of visual oddball paradigm by recording EEG. Event-related beta responses increased in power and in phase synchrony upon presentation of target stimulation in comparison to non-target stimulation. There were also contradictory results with our study. Beta ERDs upon presentation of target stimulation were reported by Cacace and McFarland (2003), Ishii et al. (2009), and Mazaheri and Picton (2005). It should also be noted that the authors of those previous studies asked subjects to press a button upon presentation of target stimuli. Therefore, in those previous studies, the decrease observed in beta responses could be movement-dependent.

Event-related beta power was higher upon presentation of target stimulation in comparison to non-target stimulation in healthy subjects; there were no differences in beta power between target stimulation versus non-target stimulation in MCI patients. If the beta oscillatory responses were related to increased attention and/or cognitive load, we could tentatively conclude that MCI patients attended to both target and non-target stimuli in a similar process. Behavioral results also showed that MCI patients were worse than healthy subjects at identifying target stimuli (Section 3.1). It seems that the non-significant difference in beta power response for target versus non-target stimulation in MCI patients might indicate an inability of MCI patients to distinguish targets from non-targets.

Upon presentation of visual oddball paradigm, occipital electrodes showed enhanced higher beta response compared to other electrode sites. This result was in good accordance with previous studies, which showed that evoked oscillatory beta activity over scalp regions was associated with projections from sensory-specific cortex. Auditory stimuli enhanced beta responses at central and temporal electrodes (Haenschel et al., 2000; Makinen et al., 2004; Peterson and Thaut, 2002), whereas visual stimuli enhanced beta responses at occipital electrodes (Senkowski et al., 2006). The present study and the previous studies also indicated that whether the stimuli were either auditory or visual, beta responses increase over right hemisphere (Haenschel et al., 2000; Huster et al., 2013; Makinen et al., 2004; Senkowski et al., 2006; Swann et al., 2009, 2012).

## 5 Conclusion

1. The presented results clearly indicate that target stimulation elicited higher event-related beta power, event-related beta-phase locking and event-related filtered beta responses than non-target stimulation at all electrode sites in healthy subjects.
2. Although evoked power, event-related beta-phase locking and event-related filtered beta responses of healthy subjects were higher than those of MCI patients, the differences were not statistically significant. However, it should also be noted that, in MCI patients there was no difference between target evoked power and non-target evoked power. This might indicate that MCI subjects experience difficulty in differentiating between target and non-target stimuli.
3. Yener and Başar (2013) asked whether it is possible to learn about cognitive impairments after application just by knowing some dynamic factors that are influenced by the disease. After analyzing different frequency bands by application of different methods in MCI and AD, it appears that clues to cognitive impairment might be identified in brain oscillations.
4. Our study showed that, upon presentation of visual oddball paradigm, occipital electrodes showed enhanced beta response compared to other electrode sites. This result is accordance with studies that showed evoked oscillatory beta activity over scalp regions was associated with projections from sensory-specific cortex.
5. The present study and previous studies also indicated that, independent of stimulus modality (auditory or visual), beta responses increase over the right hemisphere (Haenschel et al., 2000; Huster et al., 2013; Makinen et al., 2004; Senkowski et al., 2006; Swann et al., 2009, 2012).
6. According to the above-mentioned studies, beta oscillations increased upon negative emotional stimulation, upon high arousal stimulation, upon multisensory stimulation and also upon cognitive load. The common mechanism between these different stimulations might be the need for increased attention. It is also of note that, since beta oscillations are also related to sensorimotor functions, it does not seem possible to propose a general hypothesis for the functional role of beta oscillations. It is possible that, similarly to delta, theta, alpha and gamma oscillations, beta oscillations are not related only to one single function—but that these functions derive from the superpositions and super-synergy of multiple brain oscillations (Başar et al., 1999, 2001, 2006).

## Figures and Tables

**Table 1:** The group characteristics of subjects. MMSE = Mini-Mental State Examination, SD = standard deviation, NS = not significant. * represents significant result.

	Controls (*N* = 17)	MCI (*N* = 17)	p
Age (SD)	68.2 (5.6)	70.5 (5.6)	**NS**
Education (SD)	10 (5.7)	10.2 (4.3)	**NS**
MMSE (SD)	29.4 (0.8)	25.8 (2.5)	0.008*

## References

[bb0220] Albert M.S., DeKosky S.T., Dickson D., Dubois B., Feldman H.H., Fox N.C., Gamst A., Holtzman D.M., Jagust W.J., Petersen R.C., Snyder P.J., Carrillo M.C., Thies B., Phelps C.H. (2011). The diagnosis of mild cognitive impairment due to Alzheimer's disease: recommendations from the National Institute on Aging and Alzheimer's Association workgroup. Alzheimer's & Dementia.

[bb0385] Babiloni C., Babiloni F., Carducci F., Cincotti F., Cocozza G., Del Percio C., Moretti D.V., Rossini P.M. (2002). Human cortical electroencephalography (EEG) rhythms during the observation of simple aimless movements: a high-resolution EEG study. NeuroImage.

[bb0005] Başar E. (2011). Brain–Body–Mind in the Nebulous Cartesian System: A Holistic Approach by Oscillations.

[bb0190] Başar E., Güntekin B. (2008). A review of brain oscillations in cognitive disorders and the role of neurotransmitters. Brain Research.

[bb0195] Başar E., Güntekin B. (2012). A short review of alpha activity in cognitive processes and in cognitive impairment. International Journal of Psychophysiology.

[bb0145] Başar E., Schürmann M., Başar-Eroglu C., Karakaş S. (1997). Alpha oscillations in brain functioning: an integrative theory. International Journal of Psychophysiology.

[bb0150] Başar E., Başar-Eroğlu C., Karakaş S., Schürmann M. (1999). Are cognitive processes manifested in event-related gamma, alpha, theta and delta oscillations in the EEG?. Neuroscience Letters.

[bb0110] Başar E., Başar-Eroglu C., Karakaş S., Schürmann M. (2001). Gamma, alpha, delta, and theta oscillations govern cognitive processes. International Journal of Psychophysiology.

[bb0400] Başar E., Güntekin B., Öniz A. (2006). Principles of oscillatory brain dynamics and a treatise of recognition of faces and facial expressions. Progress in Brain Research.

[bb0240] Başar E., Güntekin B., Tülay E., Yener G.G. (2010). Evoked and event related coherence of Alzheimer patients manifest differentiation of sensory-cognitive networks. Brain Research.

[bb0115] Başar-Eroglu C., Başar E., Demiralp T., Schürmann M. (1992). P300-response: possible psychophysiological correlates in delta and theta frequency channels, a review. International Journal of Psychophysiology.

[bb0085] Cacace A.T., McFarland D.J. (2003). Spectral dynamics of electroencephalographic activity during auditory information processing. Hearing Research.

[bb0230] Caravaglios G., Costanzo E., Palermo F., Muscoso E.G. (2008). Decreased amplitude of auditory event-related delta responses in Alzheimer's disease. International Journal of Psychophysiology.

[bb0260] Dauwels J., Vialatte F., Cichocki A. (2010). Diagnosis of Alzheimer's disease from EEG signals: where are we standing?. Current Alzheimer Research.

[bb0280] Delorme A., Makeig S. (2004). EEGLAB: an open source toolbox for analysis of single-trial EEG dynamics including independent component analysis. Journal of Neuroscience Methods.

[bb0155] Demiralp T., Ademoglu A. (2001). Decomposition of event-related brain potentials into multiple functional components using wavelet transform. Clinical Electroencephalography.

[bb0120] Demiralp T., Ademoglu A., Schürmann M., Başar-Eroglu C., Başar E. (1999). Detection of P300 waves in single trials by the wavelet transform (WT). Brain and Language.

[bb0045] Engel A.K., Fries P. (2010). Beta-band oscillations—signalling the status quo?. Current Opinion in Neurobiology.

[bb0070] Güntekin B., Başar E. (2007). Emotional face expressions are differentiated with brain oscillations. International Journal of Psychophysiology.

[bb0355] Güntekin B., Başar E. (2007). Gender differences influence brain's beta oscillatory responses in recognition of facial expressions. Neuroscience Letters.

[bb0075] Güntekin B., Başar E. (2010). A new interpretation of P300 responses upon analysis of coherences. Cognitive Neurodynamics.

[bb0175] Güntekin B., Başar E. (2010). Event-related beta oscillations are affected by emotional eliciting stimuli. Neuroscience Letters.

[bb0245] Güntekin B., Saatçi E., Yener G.G. (2008). Decrease of evoked delta, theta and alpha coherences in Alzheimer patients during a visual oddball paradigm. Brain Research.

[bb0320] Haenschel C., Baldeweg T., Croft R.J., Whittington M., Gruzelier J. (2000). Gamma and beta frequency oscillations in response to novel auditory stimuli: a comparison of human electroencephalogram (EEG) data with in vitro models. Proceedings of the National Academy of Sciences of the United States of America.

[bb0285] Herrmann C.S., Munk M.H., Engel A.K. (2004). Cognitive functions of gamma-band activity: memory match and utilization. Trends in Cognitive Science.

[bb0050] Huster R.J., Enriquez-Geppert S., Lavallee C.F., Falkenstein M., Herrmann C.S. (2013). Electroencephalography of response inhibition tasks: functional networks and cognitive contributions. International Journal of Psychophysiology.

[bb0090] Ishii R., Canuet L., Herdman A., Gunji A., Iwase M., Takahashi H., Nakahachi T., Hirata M., Robinson S.E., Pantev C., Takeda M. (2009). Cortical oscillatory power changes during auditory oddball task revealed by spatially filtered magnetoencephalography. Clinical Neurophysiology.

[bb0255] Jeong J. (2004). EEG dynamics in patients with Alzheimer's disease. Clinical Neurophysiology.

[bb0125] Karakaş S., Erzengin O.U., Başar E. (2000). A new strategy involving multiple cognitive paradigms demonstrates that ERP components are determined by the superposition of oscillatory responses. Clinical Neurophysiology.

[bb0030] Karrasch M., Laine M., Rinne J.O., Rapinoja P., Sinervä E., Krause C.M. (2006). Brain oscillatory responses to an auditory–verbal working memory task in mild cognitive impairment and Alzheimer's disease. International Journal of Psychophysiology.

[bb0325] Kisley M.A., Cornwell Z.M. (2006). Gamma and beta neural activity evoked during a sensory gating paradigm: effects of auditory, somatosensory and cross-modal stimulation. Clinical Neurophysiology.

[bb0130] Kolev V., Demiralp T., Yordanova J., Ademoglu A., Isoglu-Alkaç U. (1997). Time–frequency analysis reveals multiple functional components during oddball P300. Neuroreport.

[bb0095] Kukleta M., Bob P., Brázdil M., Roman R., Rektor I. (2009). Beta 2-band synchronization during a visual oddball task. Physiological Research.

[bb0380] Kukleta M., Brázdil M., Roman R., Bob P., Rektor I. (2009). Cognitive network interactions and beta 2 coherence in processing non-target stimuli in visual oddball task. Physiological Research.

[bb0035] Kurimoto R., Ishii R., Canuet L., Ikezawa K., Iwase M., Azechi M., Aoki Y., Ikeda S., Yoshida T., Takahashi H., Nakahachi T., Kazui H., Takeda M. (2012). Induced oscillatory responses during the Sternberg's visual memory task in patients with Alzheimer's disease and mild cognitive impairment. NeuroImage.

[bb0250] Kurt P., Emek D.D., Batum K., Gölbaşı B.T., Güntekin B., Karşıdağ S., Başar E., Yener G.G. (2012). Auditory event-related delta oscillatory responses are reduced in patients with mild cognitive impairment. Biological Psychiatry.

[bb0290] Lenz D., Krauel K., Flechtner H.H., Schadow J., Hinrichs H., Herrmann C.S. (2010). Altered evoked gamma-band responses reveal impaired early visual processing in ADHD children. Neuropsychologia.

[bb0295] Lenz D., Fischer S., Schadow J., Bogerts B., Herrmann C.S. (2011). Altered evoked γ-band responses as a neurophysiological marker of schizophrenia?. International Journal of Psychophysiology.

[bb0330] Leventhal D.K., Gage G.J., Schmidt R., Pettibone J.R., Case A.C., Berke J.D. (2012). Basal ganglia beta oscillations accompany cue utilization. Neuron.

[bb0065] Lopes da Silva F., Van Rotterdam A., Storm van Leeuwen W., Tielen A.M. (1970). Dynamic characteristics of visual evoked potentials in the dog. II. Beta frequency selectivity in evoked potentials and background activity. Electroencephalography and Clinical Neurophysiology.

[bb0335] Makinen V.T., May P.J., Tiitinen H. (2004). Human auditory event-related processes in the time–frequency plane. Neuroreport.

[bb0100] Mazaheri A., Picton T.W. (2005). EEG spectral dynamics during discrimination of auditory and visual targets. Brain Research. Cognitive Brain Research.

[bb0360] Miskovic V., Schmidt L.A. (2010). Cross-regional cortical synchronization during affective image viewing. Brain Research.

[bb0040] Missonnier P., Deiber M.P., Gold G., Herrmann F.R., Millet P., Michon A., Fazio-Costa L., Ibañez V., Giannakopoulos P. (2007). Working memory load-related electroencephalographic parameters can differentiate progressive from stable mild cognitive impairment. Neuroscience.

[bb0310] Neuper C., Scherer R., Wriessnegger S., Pfurtscheller G. (2009). Motor imagery and action observation: modulation of sensorimotor brain rhythms during mental control of a brain–computer interface. Clinical Neurophysiology.

[bb0165] Öniz A., Başar E. (2009). Prolongation of alpha oscillations in auditory oddball paradigm. International Journal of Psychophysiology.

[bb0370] Onton J., Delorme A., Makeig S. (2005). Frontal midline EEG dynamics during working memory. NeuroImage.

[bb0200] Özerdem A., Güntekin B., Tunca Z., Başar E. (2008). Brain oscillatory responses in patients with bipolar disorder manic episode before and after valproate treatment. Brain Research.

[bb0205] Özerdem A., Güntekin B., Saatçi E., Tunca Z., Başar E. (2010). Disturbance in long distance gamma coherence in bipolar disorder. Progress in Neuro-Psychopharmacology & Biological Psychiatry.

[bb0210] Özerdem A., Güntekin B., Atagün M.İ., Turp-Gölbaşı B., Başar E. (2011). Reduced long distance gamma (28–48 Hz) coherence in euthymic patients with bipolar disorder. Journal of Affective Disorders.

[bb0215] Özerdem A., Güntekin B., Atagün M.İ., Başar E. (2013). Chapter 14: Brain oscillations in bipolar disorder in search of new biomarkers. Supplements to Clinical Neurophysiology.

[bb0270] Petersen R.C., Smith G.E., Waring S.C., Ivnik R.J., Tangalos E.G., Komken E. (1999). Mild cognitive impairment clinical characterization and outcome. Archives of Neurology.

[bb0340] Peterson D.A., Thaut M.H. (2002). Delay modulates spectral correlates in the human EEG of non-verbal auditory working memory. Neuroscience Letters.

[bb0305] Pfurtscheller G., Berghold A. (1989). Patterns of cortical activation during planning of voluntary movement. Electroencephalography and Clinical Neurophysiology.

[bb0055] Pfurtscheller G., Stancák A., Neuper C. (1996). Post-movement beta synchronization. A correlate of an idling motor area?. Electroencephalography and Clinical Neurophysiology.

[bb0105] Polich J., Kok A. (1995). Cognitive and biological determinants of P300: an integrative review. Biological Psychology.

[bb0235] Polikar R., Topalis A., Green D., Kounios J., Clark C.M. (2007). Comparative multiresolution wavelet analysis of ERP spectral bands using an ensemble of classifiers approach for early diagnosis of Alzheimer's disease. Computers in Biology and Medicine.

[bb0375] Ravizza S.M., Behrmann M., Fiez J.A. (2005). Right parietal contributions to verbal working memory: spatial or executive?. Neuropsychologia.

[bb0350] Sakowitz O.W., Quiroga R.Q., Schürmann M., Başar E. (2005). Spatio-temporal frequency characteristics of intersensory components in audiovisual evoked potentials. Brain Research. Cognitive Brain Research.

[bb0345] Senkowski D., Molholm S., Gomez-Ramirez M., Foxe J.J. (2006). Oscillatory beta activity predicts response speed during a multisensory audiovisual reaction time task: a high-density electrical mapping study. Cerebral Cortex.

[bb0135] Spencer K.M., Polich J. (1999). Poststimulus EEG spectral analysis and P300: attention, task, and probability. Psychophysiology.

[bb0160] Stampfer H., Başar E. (1985). Does frequency-analysis lead to better understanding of endogenous evoked-potentials. International Journal of Neuroscience.

[bb0390] Swann N., Tandon N., Canolty R., Ellmore T.M., McEvoy L.K., Dreyer S., DiSano M., Aron A.R. (2009). Intracranial EEG reveals a time- and frequency-specific role for the right inferior frontal gyrus and primary motor cortex in stopping initiated responses. Journal of Neuroscience.

[bb0395] Swann N.C., Cai W., Conner C.R., Pieters T.A., Claffey M.P., George J.S., Aron A.R., Tandon N. (2012). Roles for the pre-supplementary motor area and the right inferior frontal gyrus in stopping action: electrophysiological responses and functional and structural connectivity. NeuroImage.

[bb0300] Tallon-Baudry C., Bertrand O., Delpuech C., Pernier J. (1996). Stimulus specificity of phase-locked and non-phase-locked 40 Hz visual responses in human. Journal of Neuroscience.

[bb0365] Tallon-Baudry C., Bertrand O., Peronnet F., Pernier J. (1998). Induced gamma-band activity during the delay of a visual short-term memory task in humans. Journal of Neuroscience.

[bb0315] Traub R.D., Whittington M.A., Buhl E.H., Jefferys J.G., Faulkner H.J. (1999). On the mechanism of the gamma → beta frequency shift in neuronal oscillations induced in rat hippocampal slices by tetanic stimulation. Journal of Neuroscience.

[bb0080] Woodruff C.C., Daut R., Brower M., Bragg A. (2011). Electroencephalographic alpha-band and beta-band correlates of perspective-taking and personal distress. Neuroreport.

[bb0060] Wróbel A. (2000). Beta activity: a carrier for visual attention. Acta neurobiologiae Experimentalis.

[bb0185] Yener G.G., Başar E. (2010). Sensory evoked and event related oscillations in Alzheimer's disease: a short review. Cognitive Neurodynamics.

[bb0025] Yener G.G., Başar E. (2013). Biomarkers in AD with a special emphasis on event-related oscillatory responses. Supplements to Clinical Neurophysiology.

[bb0010] Yener G.G., Güntekin B., Öniz A., Başar E. (2007). Increased frontal phase-locking of event-related theta oscillations in Alzheimer patients treated with cholinesterase inhibitors. International Journal of Psychophysiology.

[bb0015] Yener G.G., Güntekin B., Başar E. (2008). Event-related delta oscillatory responses of Alzheimer patients. European Journal of Neurology.

[bb0020] Yener G.G., Güntekin B., Tülay E., Başar E. (2009). A comparative analysis of sensory visual evoked oscillations with visual cognitive event related oscillations in Alzheimer's disease. Neuroscience Letters.

[bb0225] Yener G.G., Güntekin B., Örken D.N., Tülay E., Forta H., Başar E. (2012). Auditory delta event-related oscillatory responses are decreased in Alzheimer's disease. Behavioural Neurology.

[bb0265] Yener G.G., Kurt P., Emek D.D., Güntekin B., Başar E. (2013). Reduced visual event-related delta oscillatory responses in amnestic mild cognitive impairment. Journal of Alzheimer's Disease.

[bb0275] Yesavage J.A., Brink T.L., Rose T.L., Lum O., Huang V., Adey M., Leirer V.O. (1983). Development and validation of a geriatric depression screening scale: a preliminary report. Journal of Psychiatric Research.

[bb0170] Yordanova J., Kolev V. (1998). A single-sweep analysis of the theta frequency band during an auditory oddball task. Psychophysiology.

[bb0140] Yordanova J., Devrim M., Kolev V., Ademoglu A., Demiralp T. (2000). Multiple time–frequency components account for the complex functional reactivity of P300. Neuroreport.

